# Transcriptional profiling of methyl jasmonate-induced defense responses in bilberry (*Vaccinium myrtillus* L.)

**DOI:** 10.1186/s12870-019-1650-0

**Published:** 2019-02-12

**Authors:** Rafael Fonseca Benevenuto, Tarald Seldal, Stein Joar Hegland, Cesar Rodriguez-Saona, Joseph Kawash, James Polashock

**Affiliations:** 1 Faculty of Engineering and Science, Western Norway University of Applied Sciences, Sogndal, Norway; 2 Faculty of Environmental Sciences and Natural Resource Management, Norwegian University of Life Sciences, Ås, Norway; 3 Rutgers, Department of Entomology, Philip E. Marucci Center for Blueberry and Cranberry Research, The State University of New Jersey, Chatsworth, NJ USA; 4 Genetic Improvement of Fruits and Vegetables Lab, Philip E. Marucci Center for Blueberry and Cranberry Research, United States Department of Agriculture-Agricultural Research Service, Chatsworth, NJ USA

**Keywords:** Differential expression, Transcriptome, Herbivory, Secondary metabolism, Flowering, Signaling

## Abstract

**Background:**

Bilberry (*Vaccinium myrtillus* L.) is one of the most abundant wild berries in the Northern European ecosystems. This species plays an important ecological role as a food source for many vertebrate and invertebrate herbivores. It is also well-recognized for its bioactive compounds, particularly substances involved in natural defenses against herbivory. These defenses are known to be initiated by leaf damage (e.g. chewing by insects) and mediated by activation of the jasmonic acid (JA) signaling pathway. This pathway can be activated by exogenous application of methyl jasmonate (MeJA), the volatile derivative of JA, which is often used to stimulate plant defense responses in studies of plant-herbivore interactions at ecological, biochemical, and molecular organismal levels. As a proxy for herbivore damage, wild *V. myrtillus* plants were treated in the field with MeJA and changes in gene expression were compared to untreated plants.

**Results:**

The de novo transcriptome assembly consisted of 231,887 unigenes. Nearly 71% of the unigenes were annotated in at least one of the databases interrogated. Differentially expressed genes (DEGs), between MeJA-treated and untreated control bilberry plants were identified using DESeq. A total of 3590 DEGs were identified between the treated and control plants, with 2013 DEGs upregulated and 1577 downregulated. The majority of the DEGs identified were associated with primary and secondary metabolism pathways in plants. DEGs associated with growth (e.g. those encoding photosynthesis-related components) and reproduction (e.g. flowering control genes) were frequently down-regulated while those associated with defense (e.g. encoding enzymes involved in biosynthesis of flavonoids, lignin compounds, and deterrent/repellent volatile organic compounds) were up-regulated in the MeJA treated plants.

**Conclusions:**

Ecological studies are often limited by controlled conditions to reduce the impact of environmental effects. The results from this study support the hypothesis that bilberry plants, growing in natural conditions, shift resources from growth and reproduction to defenses while in a MeJA-induced state, as when under insect attack. This study highlights the occurrence of this trade-off at the transcriptional level in a realistic field scenario and supports published field observations wherein plant growth is retarded and defenses are upregulated.

## Background

Bilberry (*Vaccinium myrtillus* L.), also known as European blueberry, is one of the most abundant wild berries in the Northern European ecosystems. This is a long-lived deciduous clonal shrub, with evergreen stems usually 10–60 cm tall occurring mainly in the Eurasiatic boreal zone where it regularly constitutes about 40% of the ground cover [1]. Bilberry plays an important ecological role as food source for many species of vertebrate and invertebrate herbivores, pollinators, and fruit-eating birds and mammals in the boreal ecosystems [2–8]. This species is also well recognized for its bioactive properties and has attracted worldwide interest for being considered as one of the best sources of phenolic compounds, especially anthocyanins and other flavonoids [9–11]. From an ecological standpoint, such phenolic compounds and other secondary metabolites are known to play both direct and indirect roles in plant defense against biotic and abiotic stresses. For instance, some secondary metabolites are volatile organic compounds (VOCs), which can directly deter herbivores and/or provide insects with oviposition and feeding cues [12, 13]. These compounds can also act indirectly as chemical defenses by recruiting natural enemies [14, 15].

Jasmonic acid (JA), and its VOC analog methyl jasmonate (MeJA) are signaling molecules produced by plants, especially when subjected to environmental stresses such as wounding or pathogen attack. Once the plant perceives JA signals, a considerable reprogramming of gene expression occurs. Consequently, changes in the regulation of important pathways are made, including the induction of defensive genes and their associated biosynthetic pathways [16]. Inducible defense responses in plants can also be activated by exogenous application of MeJA. These responses to MeJA are similar to those induced by natural induction and include production of a range of toxic metabolites and anti-digestive proteins, such as proteinase inhibitors, which harm both specialist and generalist herbivores [17]. Studies in *Nicotiana attenuata* plants showed that trypsin proteinase inhibitor activity increased after MeJA elicitation [18]. As MeJA-induced responses are generally similar to those induced by insect herbivory [19], the application of exogenous MeJA is a useful tool to stimulate plant resistance in studies of plant-herbivore interactions at multiple organismal levels.

Recent ecological studies have reported significant changes in bilberry plants induced with MeJA treatment in their natural environments, including significant reduction in insect herbivory and plant growth [3, 20–22]. These studies have documented an apparent trade-off between growth/reproduction and defense in bilberry plants. However, little is known about the global changes in gene expression in induced plants to optimize their resource allocations from growth/reproduction to defense. Mayrose et al. [23] identified several genes, including a protein phosphatase 2C and the HD-Zip transcription factor Athb-8, whose expression are associated with trade-offs between growth and defense in common sunflower (*Helianthus annuus*). Similarly, Mitra and Baldwin [24] showed that RuBPCase activase, an abundant photosynthetic protein, mediates growth-defense trade-offs in *N. attenuata* by attenuating JA-induced defenses. However, changes in bilberry plants at the transcriptome level in response to defense induction by using MeJA have not yet been reported, though such changes must be a prerequisite to induce the synthesis of defensive metabolites and proteins related to plant defense [25].

The aim of our current work is to report, for the first time, a comprehensive transcriptome profile of MeJA-induced bilberry plants as compared to untreated control plants. Since defense- induced bilberry plants display an effective trade-off between growth/reproduction and defense [21, 22], we predict finding supporting evidence at the transcriptional level. Specifically, we expect to find down-regulation of important genes involved in growth and reproduction and up-regulation of defense-related genes. From this, we aim to provide a catalog of the primary genes, including those that are differentially expressed, involved in growth, reproduction and plant defense pathways of this ecologically important species in the boreal ecosystem.

## Results

### Transcriptome assembly, functional annotation, and gene expression

An average of 71,799,744 reads were generated for MeJA-treated samples after filtering, with 95.37% > Q20 and 46.1% GC content. Control samples generated 62,801,772 reads after filtering, 95.49% > Q20 and 46.3% GC content. The de novo transcriptome assembly consisted of 231,887 unigenes. Among the total assembled unigenes, 60,519 were 500 bp – 1 kbp, 51,029 were 1–2 kbp and 42,432 were ≥ 2 kbp, with a mean unigene length of 1226 bp and an N50 of 1987 bp. Of the 231,877 unigenes detected, 164,262 or 70.83%, were annotated in at least one of the databases interrogated.

**Fig. 1:**
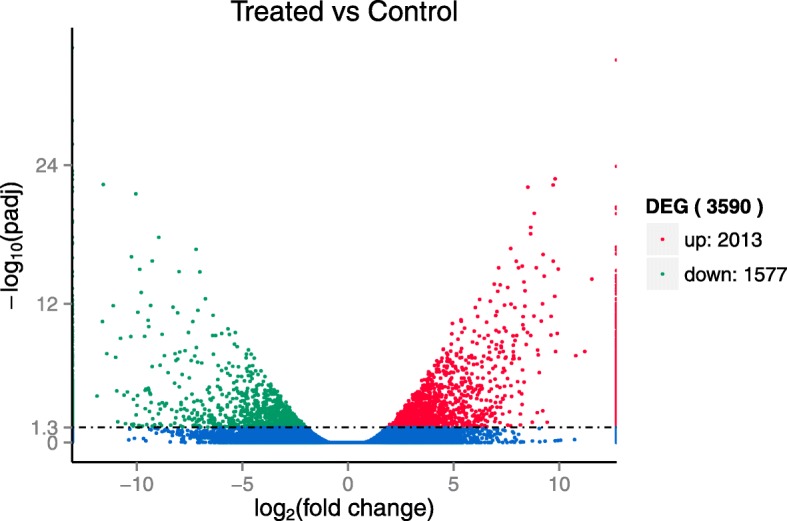
Volcano plot of overall distribution of DEGs between Treated vs Control. The x-axis shows the log_2_fold change in gene expression between treated and control samples. The y-axis shows the -log_10_ (normalized pvalue) of the difference in expression. The further from 0 on the x-axis, the greater the change in expression and the higher on the y-axis, the greater the significance

For expression analysis, the de novo transcriptome filtered by Corset was used as the reference and nearly 80% of all reads mapped back to the reference. The fragments per kilobase of exon per million fragments mapped (FPKM) distribution showed that overall expression levels were similar between the MeJA plants and the untreated controls. A total of 3590 DEGs were identified between control and MeJA-treated bilberry plants with 2013 DEGs being up regulated and 1577 being down regulated (Fig. 1).

**Fig. 2:**
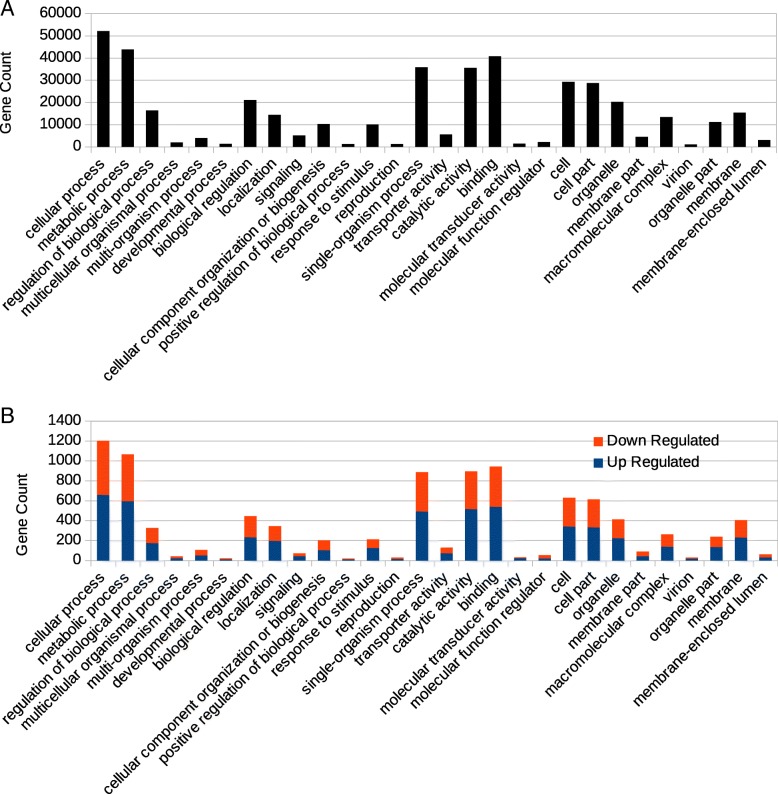
GO Classification of Differentially Expressed Unigenes. Total gene counts of GO terms associated with all unigenes (**a**) and differentially expressed unigenes (**b**). Up-regulated unigenes are in blue and down-regulated unigenes are in red

Predicted genes were analyzed with Blast2GO for Gene Ontology (GO) classification and grouped into three main GO domains: Biological Process (BP), Cellular Component (CG) and Molecular Function (MF). The seven predominant GO terms were: cellular process, metabolic process, single-organism process, cell, cell part, binding and catalytic activity (Fig. 2a). All associated GO term categories contained several differentially expressed genes, both upregulated and downregulated (Fig. 2b). The significantly enriched GO terms in DEGs were oxidation-reduction process and single-organism metabolic process, both within the GO domain of BP, as well as oxidoreductase activity in the GO domain of MF.

**Fig. 3:**
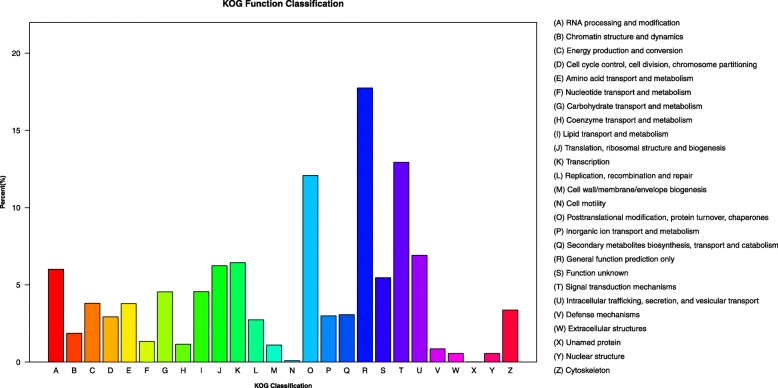
KOG function classification of all unigenes

**Fig. 4:**
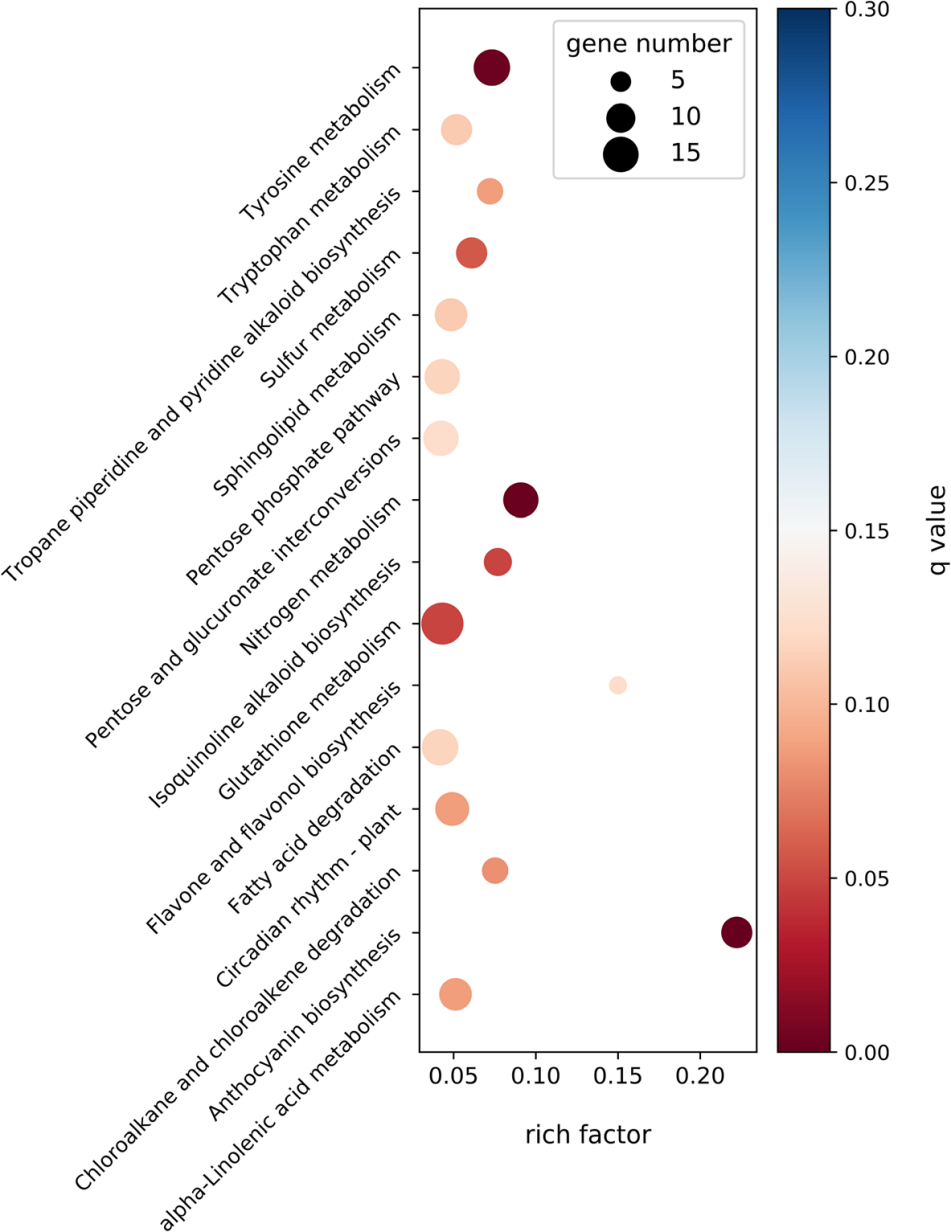
KEGG Enrichment Scatter Plot of top 16 enriched pathways. KEGG enrichment scattered plot shows the DEGs enrichment analysis results in KEGG pathway. The top 16 significantly DEGs enriched pathways are displayed in the report (padj < 0.05). The degree of KEGG enrichment is measured by rich factor (x-axis), described as the ratio of the number of DEGs in the pathway compared to the total number of genes found in the pathway. The q-value is the *p*-value adjusted to sample distribution (also referred to as padj) and indicates significance of pathway enrichment. Dot size represents the number of different genes and the color indicates the q-value

The annotated sequences were categorized into clusters of eukaryotic orthologous groups (KOG) classifications. In a total of 26 KOG categories, general function prediction only showed to be the largest group, followed by signal transduction mechanisms and posttranslational modification, protein turnover and chaperones (Fig. 3). Lastly, aiming to understand the biological pathways activated in MeJA-treated bilberry and its untreated control, all unigenes were mapped against the Kyoto Encyclopedia of Genes and Genomes (KEGG) database and 49,627 were annotated to 129 different KEGG pathways. The most represented pathways were translation, carbohydrate metabolism and folding, sorting and degradation. Mapping the DEGs in the KEGG database revealed that the pathways with the most significant changes in response to MeJA treatment were anthocyanin biosynthesis, nitrogen metabolism, tyrosine metabolism, and glutathione metabolism (Fig. 4).

### Pathways with differentially expressed genes

#### Plant hormone signaling

Groups of genes involved in different plant hormone signaling pathways, such as abscisic acid (ABA), auxin (AUX), salicylic acid (SA), ethylene (ET) and brassinosteroid (BR), were identified as differentially expressed in MeJA-treated bilberry plants compared to the water/ethanol-treated control. MeJA treatment induced genes related to ABA, ET and BR signaling pathways, while genes involved in AUX and SA pathways were repressed.

**Fig. 5:**
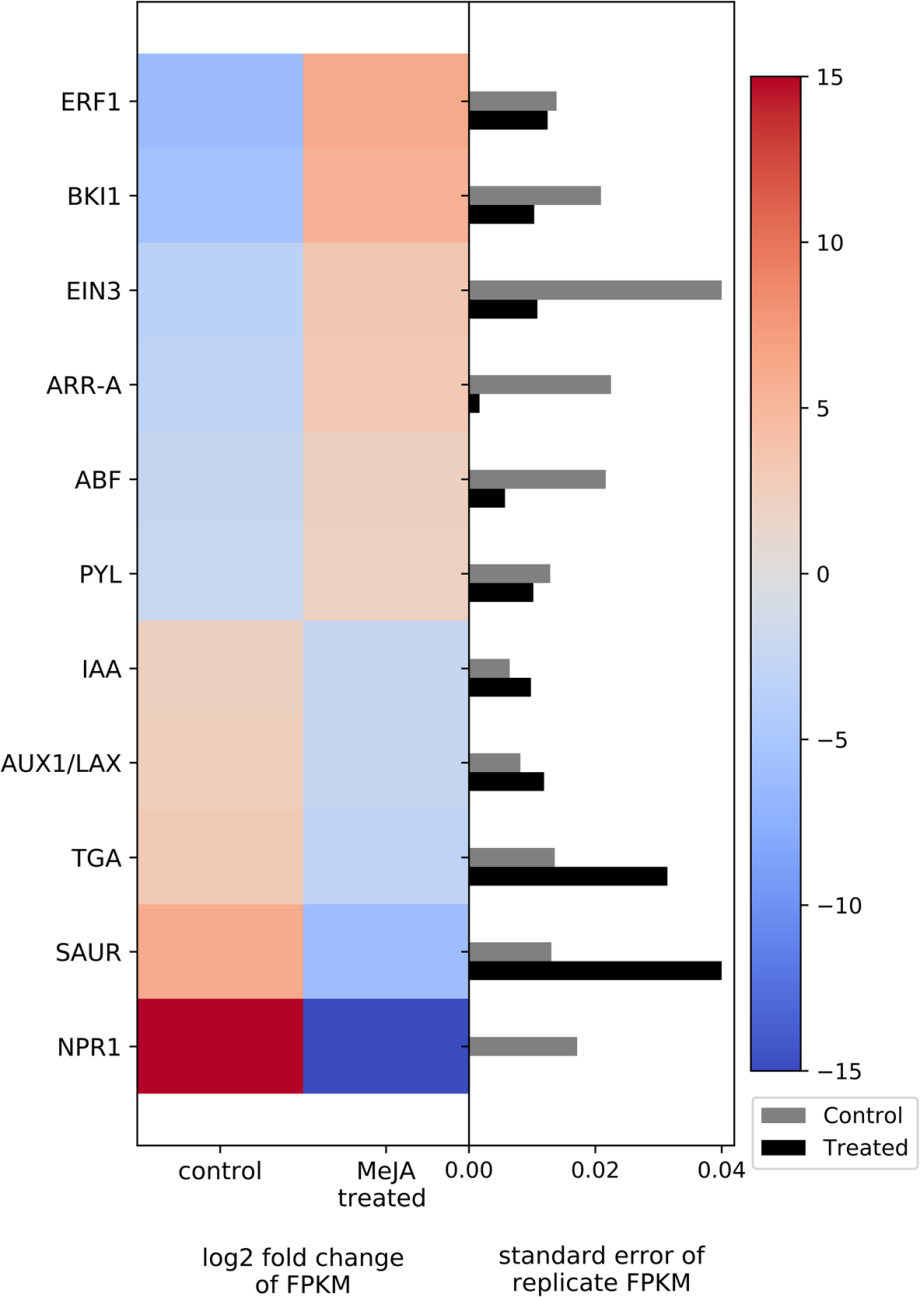
Heat map of DEGs related to hormone signaling transduction. The left side of the plot is the heatmap of log2 fold change of FPKM between all treated and control groups. The right side of the plot is the standard error for all replicates divided by the average FPKM for the control and treated groups

In the MeJA treated plants, two important genes in the ABA signaling pathway were found to be up-regulated— those encoding the PYL ABA-receptor and ABA-responsive element binding factor (*PYL* and *ABF* genes, respectively) (Fig. 5). Two genes in the ET signaling pathway— encoding ET-insensitive protein 3 (*EIN3* gene) and ET-responsive transcription factor 1 (*ERF1* gene)—and one in the BR pathway— encoding BRI1 kinase inhibitor (*BKI1* gene) were also significantly up-regulated MeJA-treated bilberry plants. In contrast, three main groups of genes involved in the AUX signaling pathway— encoding AUX1/LAX influx carrier family (*AUX1/LAX* gene), auxin-responsive protein IAA (*IAA* gene), and Small auxin upregulated RNA protein (*SAUR* gene)— as well as two main down-regulated genes involved in the SA pathway—encoding TGA transcription factor (*TGA* gene) and *NPR1* regulatory gene — were identified as significantly down-regulated in the MeJA-treated bilberry plants (Fig. 5).

#### Secondary metabolites

**Fig. 6:**
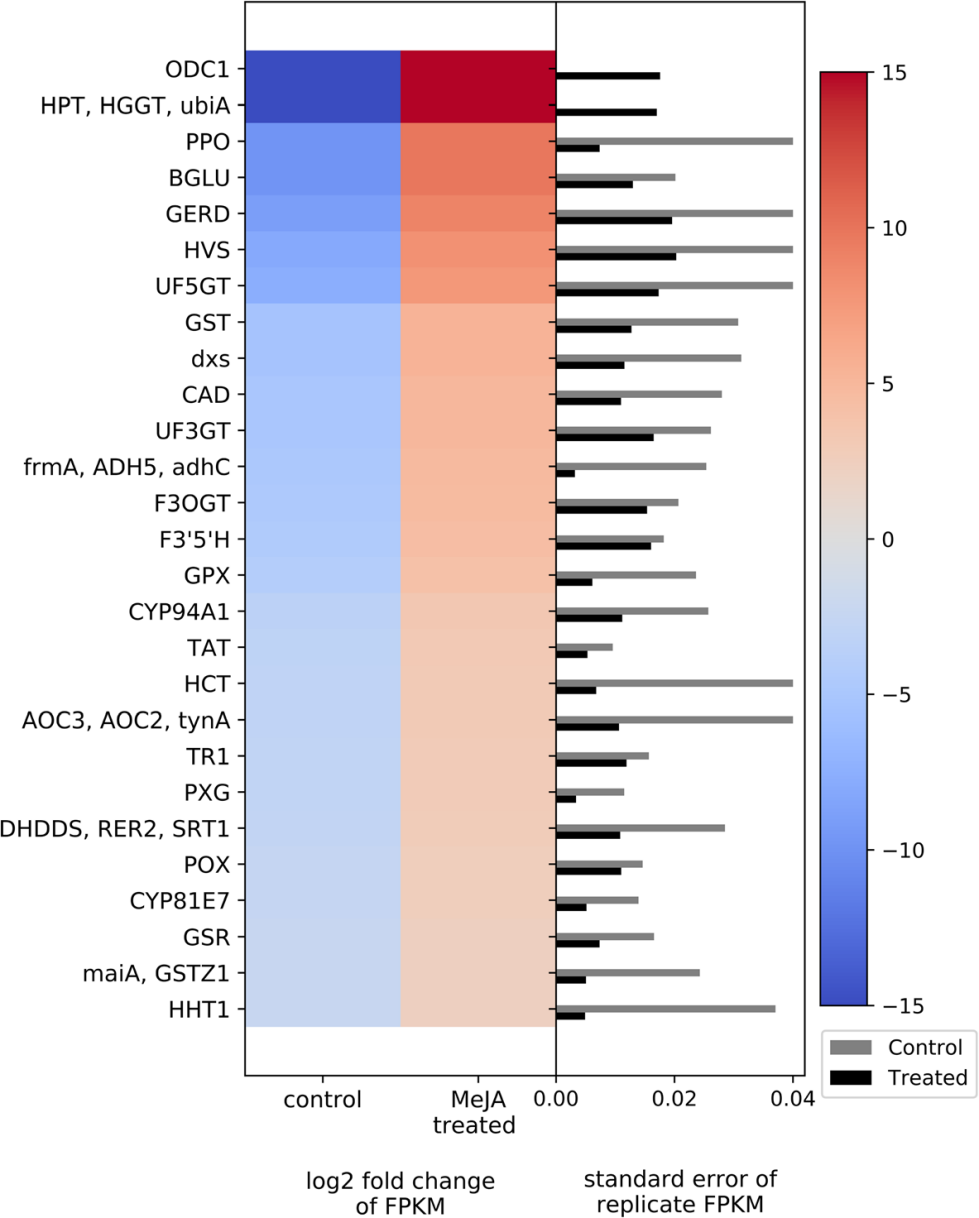
Heat map of DEGs related to secondary metabolite biosynthesis. The left side of the plot is the heatmap of log2 fold change of FPKM between all treated and control groups. The right side of the plot is the standard error for all replicates divided by the average FPKM for the control and treated groups

KEGG pathway enrichment analysis of significantly up-regulated DEGs was performed to predict what secondary metabolites might be synthesized in response to MeJA treatment of bilberry plants in the wild. We found that the anthocyanin biosynthesis pathway was the most enriched up-regulated pathway, where 9 of 45 annotated genes involved in the pathway were significantly up-regulated in response to MeJA treatment (Fig. 6). We also found DEGs in the flavone and flavonol biosynthesis pathways in MeJA-treated bilberry leaves, where two key genes in the pathway were up-regulated— those encoding flavonoid 3′,5′-hydroxylase (*F3’5’H* gene) and flavonol 3-O glucosyltransferase (*F3OGT* gene) (Fig. 6). The phenylpropanoid biosynthetic pathway appeared to be up-regulated in the MeJA induced bilberry plants (Fig. 6), as evidenced by significant up-regulation of three genes in different steps of the phenylpropanoid pathway— those encoding shikimate O-hydroxycinnamoyltransferase (*HCT* gene), cinnamyl-alcohol dehydrogenase (*CAD* gene), and peroxidase (*POX* gene) (Fig. 6).

We found the tyrosine metabolic pathway was also affected by the MeJA treatment in bilberry plants. Specifically, we found many genes in the pathway significantly up-regulated—those encoding tyrosine aminotransferase (*TAT* gene), primary-amine oxidase (*AOC3*, *AOC2*, *tynA* genes), polyphenol oxidase (*PPO* gene), alcohol dehydrogenase (*frmA*, *ADH5*, *adhC* genes), homogentisate phytyltransferase (*HPT*, *HGGT*, *ubiA* genes), and maleylacetoacetate isomerase (*maiA*, *CSTZ1* genes) (Fig. 6).

Additionally, MeJA induced genes in the glutathione metabolic pathway. The four up-regulated genes in the pathway are responsible for encoding the enzymes glutathione reductase (*GSR* gene), glutathione peroxidase (*GPX* gene), glutathione S-transferase (*GST* gene), and ornithine decarboxylase (*ODC1* gene) (Fig. 6). Finally, two terpene synthase genes that are directly involved in the synthesis of germacrene-type sesquiterpenoids were found to be up-regulated in MeJA induced plants— those encoding germacrene D synthase (*GERD* gene) and vetispiradiene synthase (*HVS* gene) (Fig. 6).

#### Circadian rhythm and flowering timing

**Fig. 7:**
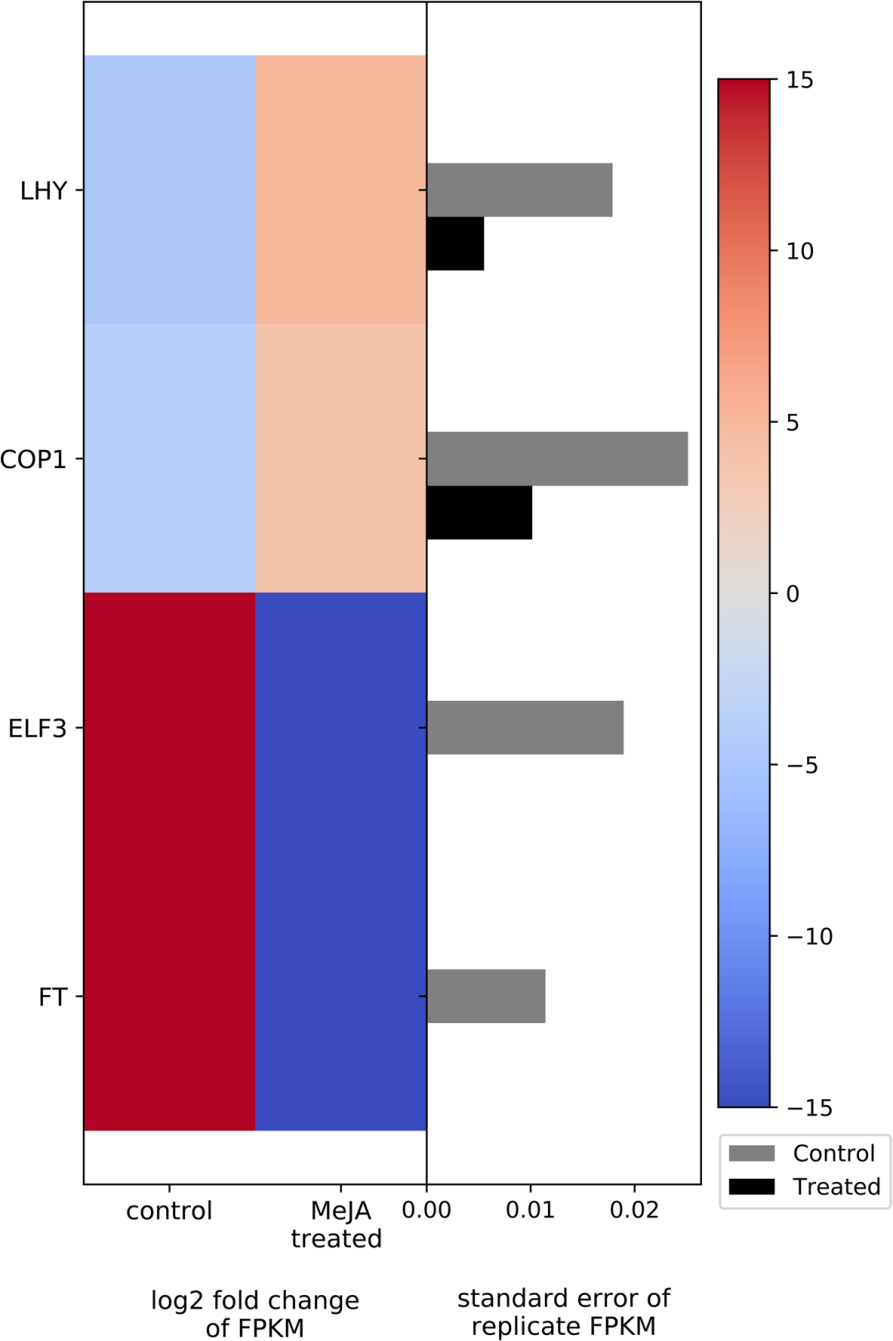
Heat map of DEGs related to circadian rhythm and flowering time. The left side of the plot is the heatmap of log2 fold change of FPKM between all treated and control groups. The right side of the plot is the standard error for all replicates divided by the average FPKM for the control and treated groups

MeJA treatment modified gene expression of the circadian clock in bilberry plants. A group of four important genes involved in circadian rhythm as well as flowering time had altered expression in response to MeJA induction. Genes encoding E3 ubiquitin-protein ligase COP1 (*COP1* gene) and MYB-related transcription factor late elongated hypocotyl (*LHY* gene) were significantly up-regulated in the MeJA-treated plants, while genes encoding the proteins early flowering 3 (*ELF3* gene) and flowering locus T (*FT* gene) were down-regulated (Fig. 7).

#### Photosynthesis and carbohydrate metabolism

**Fig. 8:**
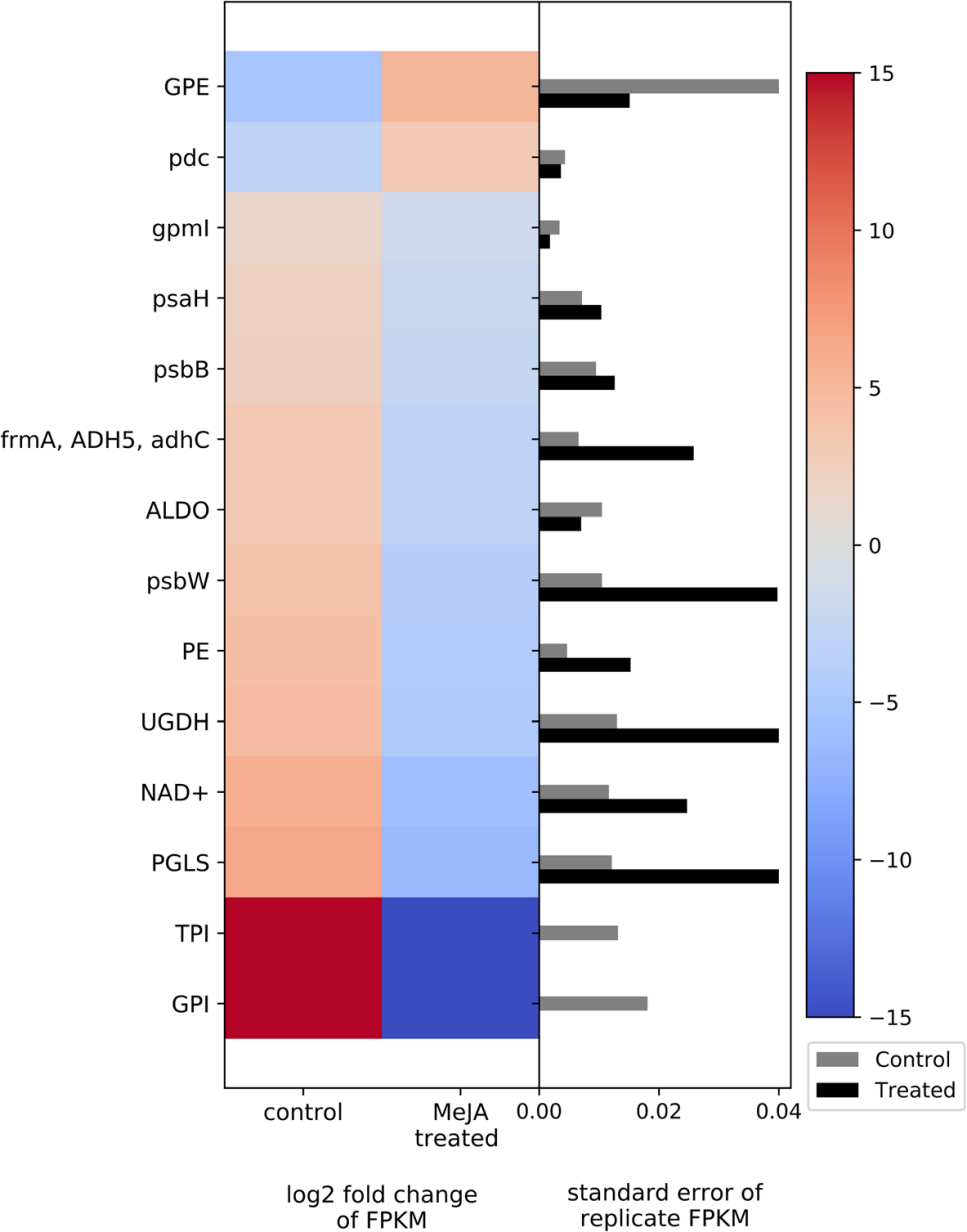
Heat map of DEGs related to photosynthesis and carbohydrate metabolism. The left side of the plot is the heatmap of log2 fold change of FPKM between all treated and control groups. The right side of the plot is the standard error for all replicates divided by the average FPKM for the control and treated groups

Plant growth is directly tied to photosynthesis and carbohydrate metabolism. To investigate the possible trade-off between growth and defense at the transcript level, we analyzed the expression of pivotal genes involved in pathways related to photosynthesis and carbohydrate metabolism in the leaves of MeJA-treated bilberry plants. Our RNA-seq results showed significant down-regulation of three genes directly involved in photosynthesis— those encoding photosystem II CP47 chlorophyll apoprotein (*psbB* gene), photosystem II psbW (*psbW* gene), and photosystem I subunit VI (*psaH* gene) (Fig. 8). Other genes involved in carbohydrate metabolism-related pathways, such as glycolysis and the pentose phosphate pathway, were also affected by the MeJA treatment— those encoding glucose-6-phosphate isomerase (*GPI* gene), glucose-6-phosphate 1-epimerase (*GPE* gene), fructose-biphosphate aldolase (*ALDO* gene), triosephosphate isomerase (*TPI* gene), 2,3-biphosphoglycerate-independent phosphoglycerate mutase (*gpmI* gene), pyruvate decarboxylase (*pdc* gene), aldehyde dehydrogenase (*NAD+* gene), alcohol dehydrogenase (*ADH5* gene), 6-phosphoglucolactonase (*PGLS* gene), pectinesterase (*PE* gene), and UDPglucose 6-dehydrogenase (*UGDH* gene) (Fig. 8).

#### Nitrogen metabolism

**Fig. 9:**
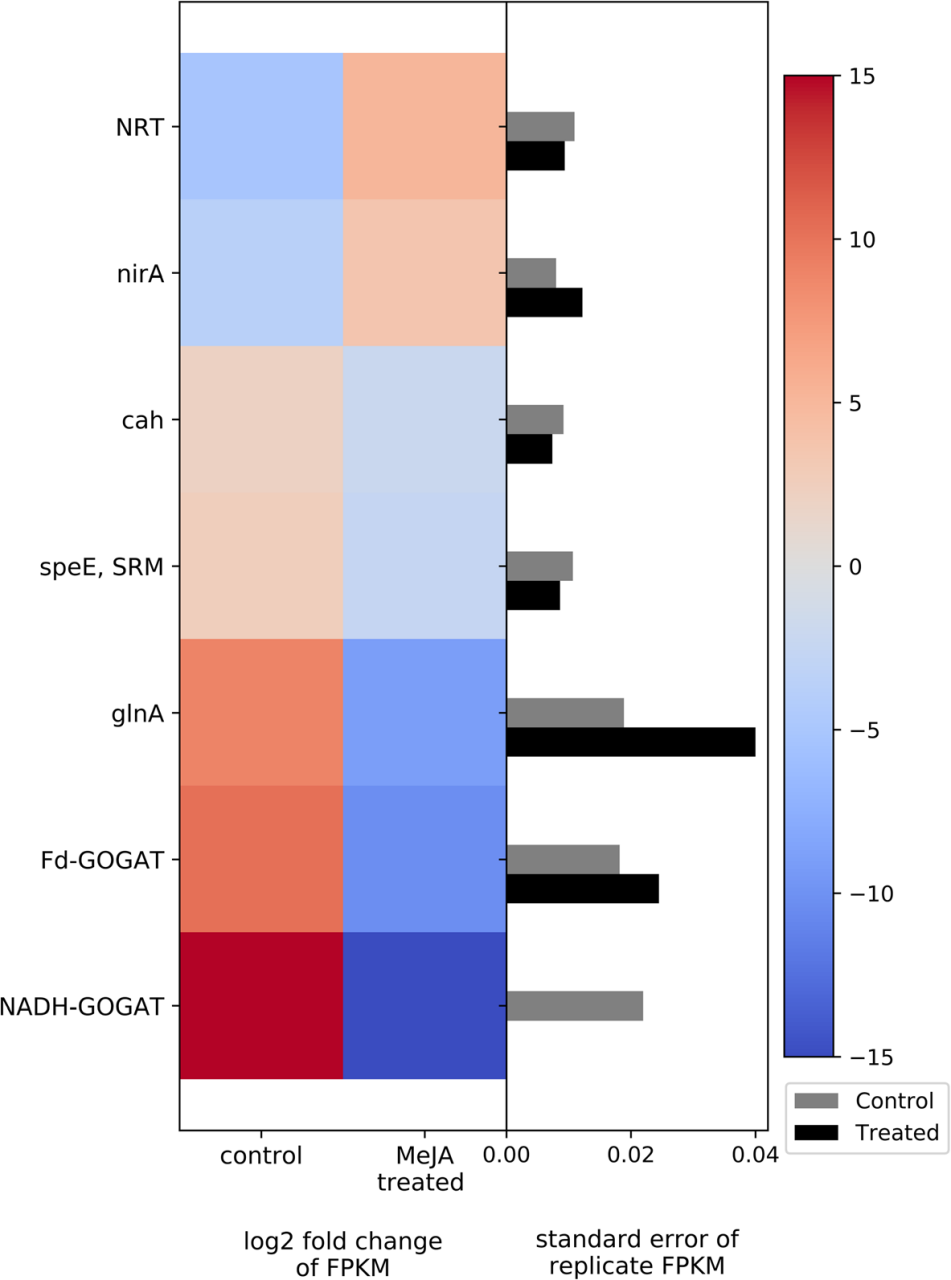
Heat map of DEGs related to nitrogen metabolism. The left side of the plot is the heatmap of log2 fold change of FPKM between all treated and control groups. The right side of the plot is the standard error for all replicates divided by the average FPKM for the control and treated groups

Nitrogen metabolism is also tied to growth and development as nitrogen is an important constituent of DNA, RNA, proteins, hormones, chlorophyll, and other critical plant compounds. We found a group of important DEGs involved in the nitrogen metabolic pathway also being affected by MeJA treatment in bilberry leaves. Two genes involved in transport and reduction of nitrite were up-regulated— those encoding NRT nitrate/nitrite transporter (*NRT* gene) and ferredoxin-nitrite reductase (*nirA* gene). Yet, expression levels of six other genes linked to glutamate metabolism were repressed— those encoding carbonic anhydrase (*cah* gene), chloroplastic glutamine synthetase (*glnA* gene), NADH-dependent glutamate synthase (*NADH-GOGAT* gene), ferredoxin-dependent glutamate synthase (*Fd-GOGAT* gene), and spermidine synthase (*speE*, *SRM* genes) (Fig. 9).

## Discussion

### Plant hormone signaling

Plant hormones operate in a complex “crosstalk” network between different pathways [26] and it is evident that MeJA treatment impacts this signaling. In the MeJA treated plants, two important genes in the ABA signaling pathway were found to be up-regulated—*PYL* and *ABF* (Fig. 5). Lackman et al. [27] showed that PYL ABA-receptor encoding genes are involved in the crosstalk between the JA and ABA signaling pathways to regulate metabolism and growth. Altered expression of PYL ABA-receptor encoding genes, *PYL4* and *PYL5*, was shown to affect JA responses, both in terms of biomass and anthocyanin production, which contributes to understanding the role of JAs in balancing the trade-off between growth and defense [27]. This trade-off has also been demonstrated in ecological studies where MeJA-treated wild bilberry plants showed consistent induction of defense responses leading to suppressed vegetative growth and reduced caterpillar herbivory, suggesting the allocation of resources from growth to defense [20, 21].

Several studies provide evidence for positive interactions between the JA and ET signaling pathways, especially regarding the regulation of defense-related genes [28–31]. MeJA-treated bilberry plants showed two significantly up-regulated genes in the ET signaling pathway— *EIN3* and *ERF1*—and one in the BR pathway—*BKI1*. *EIN3* is well documented as a JA-responsive ethylene-signaling gene [32]. Downstream in the ET signaling pathway, *ERF1*, encoding an ET-responsive transcription factor, is known to play important roles in the regulation of defenses. Lu et al. [33] identified *ERF3* in rice (*Oryza sativa*) as a gene that positively affects expression of trypsin proteinase inhibitors and mediates resistance against caterpillars.

The involvement of AUX in plant growth and development, through induction of cell division and expansion, elongation, and cell differentiation is well characterized [34–36]. We identified three main groups of genes involved in the AUX signaling pathway as significantly down-regulated in the MeJA-treated bilberry plants— *AUX1/LAX*, *IAA*, and *SAUR* (Fig. 5). Yan et al. [37] studied the effects of MeJA treatment on root growth and found that JA signaling modulates AUX signaling pathways by regulating the expression of key genes involved in AUX transport. Among other AUX transporter-related genes, such as *PIN1*, *PIN2*, *PIN3*, they also found the *AUX1/LAX* family influx carrier gene to be down-regulated in response to MeJA treatment [26, 37].

Although the interactions between JA and SA appear to be complex, several genetic studies provided evidence for an antagonist regulatory interaction between them. As such, the SA signaling pathway was shown to be negatively modulated by MeJA treatment in bilberry plants. Specifically, we found two main down-regulated genes involved in the SA pathway—*NPR1* regulatory gene and *TGA* transcription factor. Studies in tobacco showed that endogenous and exogenous JA inhibit the expression of SA-dependent genes [38, 39]. The benefits and potential costs of cross talk between these important signaling pathways involved in defense have been extensively discussed [40–43]. Specifically, different defense signaling pathways have evolved the capacity to cross talk and modulate each other, allowing plants the flexibility to adjust defense responses according to a specific pathogen or herbivore attack [26]. In bilberry, JA signaling appears to synergize with ABA, ET, and BR, and to antagonize SA and AUX.

### Secondary metabolites

MeJA treatment can trigger pathways involved in the biosynthesis of certain secondary metabolites, such as terpenoids, phenylpropanoids and alkaloids, as evidenced by several studies [16, 44–48]. We conducted KEGG pathway enrichment analysis of significantly up-regulated DEGs to determine what secondary metabolites might be synthesized in response to MeJA treatment of bilberry plants. Anthocyanin biosynthesis was the most affected secondary metabolite pathway in the MeJA-treated plants, where 9 of 45 annotated genes in the pathway were significantly up-regulated in response to the MeJA treatment (Fig. 6). Anthocyanins, a class of flavonoid, are pigments present in fruits, leaves, and flowers of several plant species which act as insect and animal attractants and have been identified as an important component in plant defense mechanisms against herbivores [49]. In addition, bilberry fruit are recognized for their high anthocyanin content as compared to those of other *Vaccinium* species. Several studies have shown the health benefits of anthocyanins, especially with respect to their antioxidant activity [50–52], but there are few studies regarding the ecological importance of this secondary metabolite in the context of plant defense in these species. Although the specific role(s) of anthocyanins in plant defense is not known, the genes up-regulated in response to MeJA treatment include— genes encoding anthocyanidin 3-O-glucosyltransferase and anthocyanidin 3-O-glucoside 5-O-glucosyltransferase, also known as UDP-glucose:flavonoid 3-O-glucosyltransferase (*UF3GT* gene) and UDP-glucose:flavonoid 5-O-glucosyltransferase (*UF5GT* gene) respectively (Fig. 6). In the anthocyanin biosynthetic pathway, these genes encode key enzymes responsible for the glucosylation of anthocyanidins (i.e. pelargonidin, cyanidin, and delphinidin) to produce stable molecules [53–55].

It is well documented that flavonoids are secondary metabolites that protect plants against pathogens and herbivores, and according to the phytochemical co-evolution theory, these are important mediators of plant-insect interactions [56]. For instance, Samac and Graham [57] found a sharp and rapid up-regulation of genes encoding enzymes in the synthesis of flavone and flavonol biosynthesis by analyzing the transcriptome profile of pathogen-infected soybean (*Glycine max*) and *Medicago truncatula*. In this study, two key genes in the flavonoid pathway were found to be up-regulated in the treated plants— *F3’5’H* and *F3OGT* (Fig. 6). These genes encode enzymes that catalyze reactions for the synthesis of the flavones and flavonols: quercetins, kaempferols, syringetins, and luteolins.

The phenylpropanoid biosynthesis pathway was also up-regulated in the MeJA induced bilberry plants as indicated by up-regulation of three genes at different steps of the phenylpropanoid pathway—*HCT*, *CAD*, and *POX* (Fig. 6). *HCT* produces a transferase enzyme that catalyzes a reaction of p-Coumaroyl CoA to form intermediate substrates for the formation of important phenylpropanoids including caffeol-CoA. p-Coumaroyl CoA is an important precursor of different classes of secondary metabolites involved in plant defenses such as isoflavonoids, anthocyanins, stilbenes, and phenylpropanoid volatiles [58]. For example, the biosynthesis of chavicol, a phenylpropene VOC acting as defensive compound and floral attractant, was shown to occur via the phenylpropanoid pathway to p-Coumaroyl CoA [59]. The second up-regulated gene, *CAD*, encodes an enzyme which catalyzes the reduction of aldehyde compounds (e.g. p-coumaraldehyde, caffeoyl-aldehyde, sinapaldehyde, etc.) to form alcohol intermediates that are important substrates of lignin biosynthesis [60]. Finally, a peroxidase gene (*POX*) was up-regulated. Peroxidase acts in the very last step of the phenylpropanoid pathway, being responsible for the formation of different molignols such as syringyl lignin, p-hydroxyphenyl lignin, and guaiacyl lignin. Previous work suggested that the constitutive activation of the jasmonic acid (JA) signaling pathway leads to increased lignin deposition [61]. Among several important functions, the deposition of different lignins in the secondary plant cell walls acts as a physical barrier against pathogen attacks [62]. Studying the transcriptional profile of Chinese yew (*Taxus chinensis*) cells, Li et al. [63] also found that MeJA treatment up-regulated the expression of genes encoding key enzymes in the phenylpropanoid metabolic pathway.

We found the tyrosine metabolic pathway was also affected by the MeJA treatment in bilberry plants. Tyrosine is an aromatic amino acid which is considered a central molecule in a diverse array of plant metabolic processes including defenses and secondary metabolite biosynthesis [64]. We found six groups of genes in the pathway significantly up-regulated— *TAT*, *AOC3/AOC2/tynA*, *PPO*, *frmA/ADH5/adhC*, *HPT/HGGT/ubiA*, and *maiA/CSTZ1* (Fig. 6). Previous work showed that *TAT* was induced by MeJA and wounding at both the RNA and protein level in *Arabidopsis thaliana* [65, 66]. The *TAT* gene codes for the first enzyme in this biosynthetic pathway, which catalyzes the reaction from tyrosine to p-hydroxyphenylpyruvate, and is known to function as a radical scavenger, thus protecting plants under biotic and abiotic stress situations [66].

Additionally, MeJA induced genes in the glutathione metabolic pathway. Glutathione is an essential metabolite involved in multiple molecular functions in plants, such as redox turnover, metabolism, and signaling [67]. Glutathione is also involved in reactions linked to plant defense responses against pathogen and herbivore attack [67–69]. The four up-regulated genes in the pathway are responsible for encoding the enzymes glutathione reductase (*GSR*), glutathione peroxidase (*GPX*), glutathione S-transferase (*GST*), and ornithine decarboxylase (*ODC1*) (Fig. 6). The first two—*GSR* and *GPX*—are directly involved in the fundamental function of glutathione in redox signaling through thiol-disulfide interactions. This is a metabolic process wherein reduced glutathione (GSH) is continuously oxidized to a disulfide form (GSSG) by glutathione peroxidase, which in turn is recycled to GSH by glutathione reductase. Then, GSH is used as a source of reduced S glutathione during the biosynthesis of secondary metabolites used in defense, detoxification, and signaling processes [67]. Previous studies have shown accumulation of glutathione in response to pathogen infection [70, 71] and similar changes have been reported in response to exogenous SA treatment [72–74]. *GST* encodes an enzyme associated with a range of biochemical and physiological functions, such as antioxidative and peroxidase activities [75, 76]. However, its main function in the glutathione metabolic pathway is the catalysis of GSH for several downstream reactions, including biosynthesis of secondary metabolites [67]. It should be noted that in addition to a role in SA response, glutathione also modulates mechanisms of response to pathogens and herbivores through the JA pathway. Xiang and Oliver [77] showed that JA induces the expression of genes encoding GSH and glutathione reductase, while Sasaki-Sekimoto et al. [78] showed the same effect for other genes involved in oxidative stress and antioxidant defense.

The release of volatile terpenes is associated with anti-herbivory and also plays important roles in pollinator and natural enemy (i.e. predators and parasitoids of herbivores) attraction as well as in interactions with the surrounding environment (i.e. plant-plant signaling) [79]. Specifically, it has been suggested that germacrene D itself has deterrent effects against herbivores, as well as repellent activity against aphids and ticks [80–82]. Germacrene D has also been reported as precursor of other sesquiterpenes such as cadinenes and selinenes [83, 84]. In the MeJA-treated bilberry leaves, two genes directly involved in the synthesis of germacrene-type sesquiterpenoids were found to be up-regulated in MeJA induced plants—*GERD* and *HVS* (Fig. 6). Thus, induced bilberry plants also invest in the synthesis of specific VOCs as part of their defense strategies to either interact with its neighboring environment by, for example, recruiting natural enemies, or they could act directly as deterrents and repellents against herbivore attack.

### Circadian rhythm and flowering timing

The transition from vegetative growth to reproduction is a remarkable ‘timed’ developmental switch which can ensure the plant’s reproductive success. For timing this event, plants utilize diverse environmental indicators, such as temperature, photoperiod and light intensity, to determine the ideal time of flowering [85]. A group of four important genes involved in the circadian rhythm and flowering time had altered expression in response to MeJA induction. *COP1* and *LHY* genes were significantly up-regulated in the MeJA-treated plants, while *ELF3* and *FT* were down-regulated (Fig. 7).

The *COP1* gene codes for E3 ubiquitin ligase COP1, which is a central regulator of light-dependent physiological processes including photomorphogenesis [86], as well as circadian oscillation and flowering transition [87, 88]. *ELF3* is a clock-associated gene which encodes a protein that acts as a transcriptional modulator, controlling the expression of flowering-time regulator genes [89]. Previous studies have shown that *COP1* represses flowering by promoting degradation of Constans (*CO*), a flowering inducer gene which encodes a protein that activates the *FT* gene [87, 90]. Another study showed that downstream of the flowering pathway, *COP1* mediates *ELF3* ubiquitination and degradation [91]. Moreover, altering *ELF3* expression can cause arrhythmic expression of important morning-specific clock-regulated transcription factors, such as MYB-related LHY [92, 93], encoded by the *LHY* gene, which in our study showed to be up-regulated in response to MeJA treatment. The overexpression of LHY-related transcription factors causes not only arrhythmicity in expression of clock-regulated genes, but also in leaf movement and hypocotyl elongation [94–96]. Some plants have the capacity of altering flowering timing as a strategy to avoid insect herbivory [97, 98]. Since jasmonates are plant hormones with diverse roles in biotic and abiotic stress tolerance, the link between JA and its derivates with circadian pathways and flowering timing have been explored. For instance, JA delayed flowering in *Arabidopsis* [99, 100], and exogenous MeJA application had similar effects on flowering timing in wheat plants [101].

Based on our results, MeJA appears to induce arrhythmicity in bilberry plants via the expression of clock-regulated genes related to flowering by inducing *COP1* and *LHY*, and repressing *ELF3* and *FT*. Since induced plant defenses are energetically costly, the plant’s fitness is presumably increased by suppressing genes associated with reproduction and activating those related with defenses in response to MeJA, resulting in switching allocation of resources from growth and reproduction to defense [20–22, 102, 103].

### Photosynthesis and carbohydrate metabolism

Previous genomic studies have shown that JA and derivatives induce the expression of genes related to defenses, oxidative stress responses, senescence, and cell wall modification, while repressing the expression of genes involved in photosynthesis and metabolism-related pathways [30, 104, 105]. We found important genes from Photosystem II, *psbB* and *psbW*, which encode a protein complex responsible for light-harvesting and chlorophyll content, being down-regulated in response to MeJA treatment in bilberry leaves. The transcript encoded by *psaH* from Photosystem I was downregulated as well. These results suggest that the inhibitory effect of MeJA on photosynthesis is effectively due to the reduction in the light-harvesting complexes and, consequently, decreases in the carbon fixation process. Down-regulation of chlorophyll-related genes and chlorophyll-protein complexes were also reported in previous studies using genomic and proteomic tools to identify molecular changes in response to JA treatment [97, 104, 106, 107]. In the presence of JA, synthesis of chloroplast proteins involved in photosynthesis is immediately decreased by negative control of translation while the transcript levels remain constant. After 12 to 24 h, the transcript levels also declined, and the corresponding proteins were degraded [108]. In our study, leaf samples were collected the day after the third of three MeJA applications. Taking this into account, our results indicate that MeJA application continues repression of these transcripts for at least 24 h. The influence of JA on the expression of this group of genes leads to typical symptoms of leaf senescence—chlorophyll destruction, protein degradation, and subsequent strong yellowing [109–111].

The regulation of carbohydrate metabolism-related genes was also affected by MeJA treatment in bilberry plants. In the glycolysis and gluconeogenesis pathways, MeJA broadly down-regulated some genes involved in the fructose biosynthesis, pentose phosphate, and the TCA cycle (e.g. *GPI*, *NAD+*, *ALDO*, etc.). Interestingly, transcripts of *pdc* and *GPE*, two genes also involved glycolysis, were up-regulated in response to MeJA. Cheng et al. [112], studying the proteome of *Arabidopsis* in response to MeJA, found similar changes in carbohydrate metabolism. Sánchez-Sampedro et al. [113] reported a reprogramming in carbohydrate metabolism, where sucrose levels decreased and glucose levels increased, in milk thistle (*Silybum marianum*) suspension cultures in response to MeJA. Thus, our results suggest that MeJA induces carbohydrate catabolism, while repressing carbohydrate anabolism. In the context of induced defenses in plants, carbohydrate catabolism has an important role providing basic carbon skeletons for the biosynthesis of some secondary metabolites, such as flavonoids [113]. The results shown here indicate that at the transcript level, MeJA application induces bilberry plants to allocate carbon resources from primary towards secondary metabolism, consistent with previous findings [112, 114, 115].

### Nitrogen metabolism

As well as decreases in photosynthesis and chlorophyll content, physiological studies show that remobilization of nitrogen (N) compounds from leaves to roots and shoots is a typical senescence-like response of plants to MeJA treatment [116, 117]. We found up-regulation in the nitrite and nitrate transporter and reduction genes in the MeJA-treated bilberry leaves, which might be a preventive strategy of defense by exporting N resources out of leaves for safeguard and storing them in other tissues away from the foraging herbivores, such as shoots and roots. Gomez et al. [118] found that MeJA treatment accelerated export of N compounds from tomato leaves and described this shuttling as a strategy of defense. While nitrate transport and reduction genes were shown to be induced by MeJA-treatment, most of the genes involved in the metabolism of glutamate were down-regulated in bilberry leaves. Chloroplastic glutamine synthetase and glutamate synthase play crucial roles in amino acid synthesis and nitrogen metabolism via assimilation of ammonium obtained from the nitrate reduction and photorespiration [119]. The majority of assimilated N in plants is invested in photosynthesis, suggesting a strong positive correlation between N assimilation and photosynthetic rate [120–122]. Our observed down-regulation of genes encoding glutamine synthetase and glutamate synthase enzymes in MeJA induced bilberry leaves agrees with the idea of allocation of nitrogen-related resources from growth and development to storage as means of conserving resources while potentially under herbivore attack.

### Genetic trade-off

We found complex transcriptional changes in MeJA induced bilberry and provided evidence for allocation of resources from growth, development, and reproduction, to defense related pathways. Transcripts of receptor and response-related genes in plant hormone signal transduction, such as ABA, ET, AUX, and SA, responded to MeJA treatment through cross talk and regulation of genes involved in growth/development (e.g. *ABA* and AUX) and defense (e.g. ET and SA). Genes encoding key enzymes in metabolic pathways involved in biosynthesis of flavonoids (i.e. anthocyanins, flavones/flavonols), lignin compounds (e.g. syringyl, guaiacyl and p-hydroxyphenyl), and deterrent/repellent VOCs (e.g. phenylpropenes, sesquiterpenes) were significantly up-regulated.

Some variation in gene expression among replicates, for both the MeJA and water/ethanol-treated control, was evident (Figs. 5, 6, 7, 8, 9). This is to be expected since the experiment was conducted in the natural environment. The genotypes of the plants likely differ and thus their response to treatment may also differ. In addition, the specific environmental stresses to which the plants are naturally exposed over the 15d treatment period likely differ. Nonetheless, the changes in the transcription regulation associated with MeJA treatment highlighted in this paper are statistically significant and support the concept of genetic trade-off.

Under natural conditions, MeJA induced bilberry plants seem to invest more in the synthesis of quantitative rather than qualitative defense compounds, thus aiming to be equally effective against both specialists and generalist herbivores. Defense-induced bilberry plants synthesize a range of phenolic compounds, from lignin to more complex flavonoids, interfering in their digestion and palatability towards potential herbivores. Although MeJA induced responses effectively activate defense-related pathways, this appeared to be costly to the plant as evidenced by the fact that important genes involved in primary metabolism, such as those associated with photosynthesis, circadian rhythm, carbohydrate and nitrogen metabolism, were down-regulated, presumably to optimize the allocation of resources towards defense. Genes involved in carbohydrate anabolism were repressed, while the ones linked to carbohydrate catabolism were induced, as a possible means to allocate C resources from primary to secondary metabolism. Genes responsible for the remobilization of N sources were up-regulated, while key enzymes playing crucial roles in glutamate metabolism through ammonium assimilation, mostly invested in photosynthesis, were down-regulated. Circadian clock genes were also affected, especially transcription factors involved in flowering time. Previous studies have presented ecological evidence that inducible plant defense responses are energetically costly, where MeJA treatment increased resistance against caterpillar feeding and reduced growth and reproduction of bilberry plants [20–22]. This study provides the first evidence of resource allocation at the transcriptional level in induced bilberry plants under natural conditions.

## Conclusions

We investigated the transcriptome of MeJA treated bilberry plants compared to water/ethanol treated controls in their natural environment. Of the 23,187 unigenes annotated, 3590 differentially expressed genes (DEG) were identified, with 2013 up-regulated, and 1577 down-regulated. Further investigation of the annotated unigenes showed a significant reprogramming at the transcriptional level, whereby MeJA induced bilberry plants generally allocated resources from primary metabolism (growth, development and reproduction) to secondary metabolism (defense). The heat-maps presented suggest changes in unifying networks of genes involved in the MeJA response. Analysis of specific genes annotated as transcription factors will help us to better understand the regulation of the underlying mechanisms of the coordinated response to MeJA as a proxy for herbivore attack. This study highlights the occurrence of genetic trade-off at the transcriptional level in a realistic field scenario and supports published field observations wherein plant growth is retarded and defenses are upregulated.

## Methods

### Plant material and MeJA treatment

**Fig. 10:**
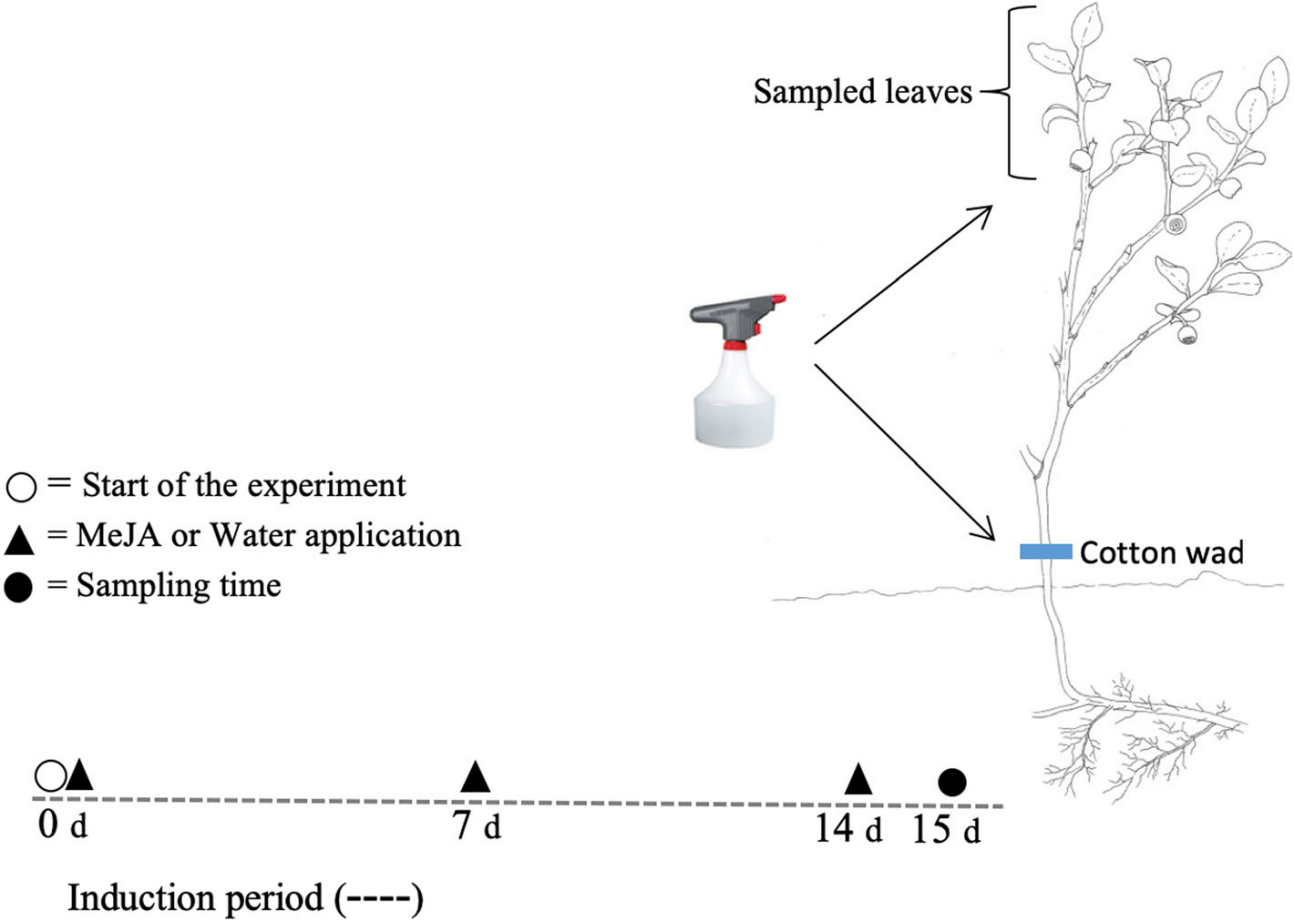
Experimental design and timeline. Bilberry plants was treated with either Methyl Jasmonate (MeJA) or Water/ethanol at 0, 7, and 14 days (d). Leaf sampling was from the most apical shoots of all plants and was performed one day after the final treatment

The experiment was conducted in a boreal forest located in Kaupanger, Western Norway (61.2^0^ N, 007.2^0^ E). The study area consisted mainly of Scots pine (*Pinus sylvestris*) with an understory dominated by bilberry and is locally recognized as one of the most important winter ranges for red deer (*Cervus elaphus*) in the inner part of the Sognefjord area. The area selected for the experiment was a 20-year-old clear-cut replanted with pines (currently ca. 150 cm tall), dominated by bilberry and other dwarf shrubs in the understory. The trees were planted ca. 2 m apart and cast relatively little shadow. In June 2016, two groups of 15 bilberry plants each were randomly selected and exposed to two treatments: 10 mM MeJA application (treated) and water/ethanol application (control). To achieve the desired concentration of MeJA, 4.1 M MeJA stock (Bedoukian Research, Danbury, CT) was diluted 1:10 with 95% (*v*/v) ethanol, and re-diluted with water to get a final concentration of 10 mM MeJA. Ethanol (95%) was added to water at the same final concentration as that in the 10 mM MeJA solution for the control. To avoid rapid evaporation of MeJA, a cotton wad was attached to the stem close to the ground and saturated with 10 mM MeJA or with water/ethanol (control). This MeJA concentration (10 mM) was shown in our earlier ecological studies to be effective in reduction of growth and levels of herbivory in bilberry [20, 21]. The applications were repeated three times at one-week intervals (Fig. 10) to simulate attack by herbivores, following the protocol used in several studies on bilberry in the field [20–22]. One day after the last treatment application, leaves from the apical part of all plants were collected and immediately frozen in liquid nitrogen and stored at − 80 °C. Samples were then transferred to RNA*later*-ICE (Life Technologies, Carlsbad, CA) and allowed to thaw at − 20 °C before RNA isolation.

### Library construction, sequencing and de novo assembly

The 15 plants in each treatment were randomly separated into five groups of three plants each. The three plants of each group were pooled and considered as one biological replicate (sample) for a total of five biological replicates per treatment for the transcriptomic analysis. Total nucleic acid was extracted from each sample using a modified CTAB procedure. Briefly, 50–70 mg of the stored leaf tissue was suspended in 800 μL cetyltrimethylammonium bromide (CTAB) buffer [123] in a 2 mL centrifuge tube with two 5 mm stainless steel beads. Samples were placed in a TissueLyserII (Qiagen, Germantown, MD) and ground for 1 min at 30 Hz. The suspension was extracted with 700 μL chloroform. After centrifugation at 11,000 *g* for 5 min, the aqueous supernatant was transferred to a new tube and total nucleic acid was precipitated by adding 0.7 volumes of isopropanol and incubating on ice for 10 min, followed by centrifugation at 13,000 *g* for 5 min. Pellets were resuspended in 400 μL of RNAse free water. Lithium chloride (100 μL of 10 M stock) was added to precipitate the total RNA and samples were incubated on ice overnight. Samples were centrifuged at 13,000 *g* for 5 min and the pellets were resuspended in 400 μL RNAse free water. The RNA was reprecipitated using ammonium acetate and ethanol. Pellets were washed with 70% ethanol and resuspended in 50 μL RNAse free water.

Libraries were constructed by Novogene Corporation (Sacramento, CA). Briefly, mRNA was enriched from total RNA using oligo (dT) beads. The mRNA was then randomly fragmented and cDNA was synthesized using random hexamers. After cDNA synthesis and library construction (terminal repair, A-tailing, ligation of sequencing adapters, size selection and PCR enrichment). The libraries were sequenced on the Illumina HiSeq platform (PE150). Raw reads were quality screened to remove adapters and those of poor quality. Clean reads were de novo assembled using Trinity [124]. The assembled transcriptome was annotated using BLAST for NR, NT, SwissProt, and KOG. For NR, NT and SwissProt databases, the evalue threshold was 1e-5 and for KOG the evalue threshold was 1e-3. PFAM, the prediction of protein structure domain: HMMER 3.0 package, hmmscan, the evalue threshold was 0.01; GO: based on the protein annotation results of NR and Pfam: Blast2GO v2.5 [125] and Novogene script, the evalue threshold was 1e-6; KEGG: KAAS, KEGG Automatic Annotation Server, the evalue threshold was 1e-10. Corset [126] was used for categorical clustering of de novo assembled contigs while individual reads were aligned with RSEM [127]. Differentially expressed genes (DEGs) between MeJA-treated and untreated control bilberry plants were identified using DESeq [128].

## Abbreviations

ABA: Abscisic acid
AUX: Auxin
BP: Biological process
BR: Brassinosteroid
CG: Cellular component
CTAB: Cetyltrimethylammonium bromide
DEGs: Differentially expressed genes
ET: Ethylene
FPKM: Fragments per kilobase of exon per million fragments mapped
GO: Gene ontology
JA: Jasmonic acid
KEGG: Kyoto Encyclopedia of Genes and Genomes
KOG: Eukaryotic Orthologous Groups
MeJA: Methyl jasmonate
MF: Molecular function
mRNA: Messenger RNA
Nr: Non-redundant protein sequences
ORFs: Open reading frames
PCR: Polymerase chain reaction
SA: Salicylic acid
VOCs: Volatile organic compounds
